# Peroxiredoxin 3 levels regulate a mitochondrial redox setpoint in malignant mesothelioma cells

**DOI:** 10.1016/j.redox.2014.11.003

**Published:** 2014-11-18

**Authors:** Brian Cunniff, Alexandra N. Wozniak, Patrick Sweeney, Kendra DeCosta, Nicholas H. Heintz

**Affiliations:** a Department of Biochemistry, University of Utah, Salt Lake City, UT, USA; b Department of Pathology, University of Vermont, College of Medicine, 149 Beaumont Avenue, Burlington, VT 05405, USA

**Keywords:** Peroxiredoxin 3, Mitochondrial structure, Cell cycle, Oxidative stress

## Abstract

Peroxiredoxin 3 (PRX3), a typical 2-Cys peroxiredoxin located exclusively in the mitochondrial matrix, is the principal peroxidase responsible for metabolizing mitochondrial hydrogen peroxide, a byproduct of cellular respiration originating from the mitochondrial electron transport chain. Mitochondrial oxidants are produced in excess in cancer cells due to oncogenic transformation and metabolic reorganization, and signals through FOXM1 and other redox-responsive factors to support a hyper-proliferative state. Over-expression of PRX3 in cancer cells has been shown to counteract oncogene-induced senescence and support tumor cell growth and survival making PRX3 a credible therapeutic target. Using malignant mesothelioma (MM) cells stably expressing shRNAs to PRX3 we show that decreased expression of PRX3 alters mitochondrial structure, function and cell cycle kinetics. As compared to control cells, knockdown of PRX3 expression increased mitochondrial membrane potential, basal ATP production, oxygen consumption and extracellular acidification rates. shPRX3 MM cells failed to progress through the cell cycle compared to wild type controls, with increased numbers of cells in G2/M phase. Diminished PRX3 expression also induced mitochondrial hyperfusion similar to the DRP1 inhibitor mdivi-1. Cell cycle progression and changes in mitochondrial networking were rescued by transient expression of either catalase or mitochondrial-targeted catalase, indicating high levels of hydrogen peroxide contribute to perturbations in mitochondrial structure and function in shPRX3 MM cells. Our results indicate that PRX3 levels establish a redox set point that permits MM cells to thrive in response to increased levels of mROS, and that perturbing the redox status governed by PRX3 impairs proliferation by altering cell cycle-dependent dynamics between mitochondrial networking and energy metabolism.

## Highlights

• Knockdown of PRX3 in malignant mesothelioma cells increases mitochondrial oxidants.
• Knockdown of PRX3 induces mitochondrial fusion and an increase in G2/M cells.
• Overexpression of catalase or mito-catalase rescues G2/M cell cycle block and mitochondrial defects.

## Graphical abstract

**Figure:**
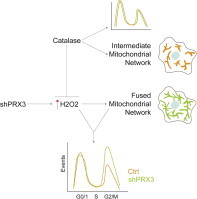

## Introduction

Oxidative stress, defined as the imbalance between the production and the elimination of cellular oxidants by antioxidants, contributes to cancer initiation, progression and survival [1]. Due to their ability to damage cellular macromolecules, reactive oxygen species (ROS) must be dynamically regulated for normal and cancer cells to maintain steady state levels below the cytotoxic threshold [1]. In normal cells oncogenic stimuli, such as activated Ras, increases the production of cellular oxidants, leading to oxidative stress and ultimately inducing senescence [2]. Tumor cells must adapt in order to evade this fate and therefore commonly over-express antioxidant enzymes, such as superoxide dismutase 2 (MnSOD, SOD2) and peroxiredoxin 3 (PRX3), which permits escape from oncogene-induced senescence [3].

Mitochondria are dynamic cellular organelles responsible for producing the majority of adenosine triphosphate (ATP), the primary energy source of the cell. Mitochondria are the primary producers of cellular ROS, both as a byproduct of aerobic respiration [4] and from other important mitochondrial sources [5]. The inner mitochondrial membrane contains the electron transport chain (ETC), which provides the driving force for ATP synthesis via electron flow, proton pumping, and the formation of an electrochemical gradient fueling ATP synthase (complex V). Electron leakage, primarily at complexes I and III, leads to the incomplete reduction of molecular oxygen which forms superoxide radical [6]. Superoxide is an unstable intermediate that is spontaneously or enzymatically dismutated to hydrogen peroxide (H_2_O_2_), the primary oxidant implicated in redox signaling [7]. Under basal conditions resident cytosolic and mitochondrial antioxidant enzymes maintain proper redox status while changes in the rate of oxidant production and metabolism activate redox-dependent signaling pathways. Numerous signaling networks responsive to cellular oxidants have been identified, and these influence survival, proliferation and stress signaling pathways in normal and pathological settings [8].

Peroxiredoxin 3 (PRX3) is a member of the typical 2-Cys peroxiredoxin family (PRX 1–4) and functions as the primary oxidoreductase in the mitochondria responsible for metabolizing H_2_O_2_ [9] . PRX3 exists as a head to tail homodimer that utilizes a peroxidatic cysteine that reacts with a molecule of H_2_O_2_, thereby forming a sulfenic acid (–SOH) intermediate. After local unfolding of the active site, the resolving cysteine located on the adjacent monomer then forms a disulfide bond with the oxidized peroxidatic cysteine [10]. Thioredoxin 2 (TRX2) reduces this disulfide bond and thereby reactivates PRX3 [11]. A structural C-terminal extension found in typical 2-cys peroxiredoxins slows disulfide bond formation, allowing another molecule of H_2_O_2_ to further oxidize the peroxidatic cysteine to sulfinic (–SO2H) acid [12]. Typically these additional oxidation events are irreversible and lead to an inactive protein, but a system comprised of sulfiredoxin and ATP specifically regenerates active PRX3 [13,14]. This is a slow, energy-dependent reaction that has been hypothesized to allow transient and local increases in ROS levels to modulate redox-dependent signaling pathways [12].

Increases in mitochondrial oxidant levels may lead to the activation of stress signaling pathways and can cause cellular damage when oxidant levels reach a cytotoxic threshold. In order to escape oxidative stress, mitochondria have been shown to undergo structural rearrangements during which damaged and healthy mitochondria fuse, effectively reducing the number of damaged mitochondrion and ameliorating oxidative stress [15]. In addition to decreasing ROS, mitochondrial fusion has been shown to support increased ATP output from mitochondria [16].

A unique form of mitochondria at the G1/S phase of the cell cycle has been identified and described as an extensive hyperfused network with higher ATP producing capacity [16]. During cell cycle progression, mitochondrial fragmentation is required for proper segregation of mitochondria to daughter cells during mitosis [17,18]. Loss of dynamin-related protein 1 (DRP1), the primary enzyme responsible for mitochondrial fission, leads to cell cycle arrest at the G2/M phase. Furthermore, loss of DRP1 leads to hyperfusion of mitochondrial networks and increased ATP levels [19]. The redox status of the cell has also been shown to play a fundamental role in cell cycle progression where transient increases in ROS activate cell cycle signaling pathways [20]. These findings provide evidence for a functional relationship between mitochondrial structure and function and cell cycle progression.

In this report we identify PRX3 as an important regulator of mitochondrial metabolism and structure in human malignant mesothelioma cells. Changes in the basal redox status of mitochondria through stable knockdown of PRX3 led to alterations in energy production and changes to the mitochondrial network through inactivation of DRP1 by dephosphorylation at Serine 616. These alterations in mitochondrial structure induced a cell cycle pause at the G2/M phase. Mitochondrial structural perturbations in shPRX3 cells, as well as cell cycle abnormalities, were rescued by expression of catalase or mito-catalase, both of which corrected mitochondrial redox status back to control levels. Together these results indicate that PRX3 establishes a mitochondrial redox set point that supports proper integration of metabolic status, mitochondrial structure and cell cycle dynamics.

## Results

### Knockdown of PRX3 increases mitochondrial oxidant levels in HM cells

**Fig. 1:**
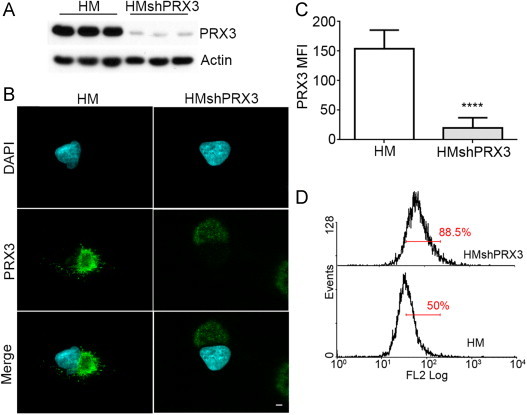
Knockdown of PRX3 in MM cells increases mitochondrial oxidant levels. (A) Immunoblot showing PRX3 expression in HM and shPRX3 HM (HMshPRX3) cell lines. Actin was used as a loading control. (B) Immunofluorescent images of HM and HMshPRX3 cells stained with anti-PRX3 antibody (green) and DAPI (cyan) (scale bar=10 µm). (C) Quantification of anti-PRX3 fluorescence intensity (mean fluorescence intensity, MFI) in HM and HMshPRX3 cells (^⁎⁎⁎⁎^*p*<0.0001, *n*=10 cells). (D) Flow cytometry histograms of HM and HMshPRX3 cells stained with MitoSOX Red; fluorescent intensity was collected in the FL2 channel. Percent cells included in the gate are indicated (red line).

Using human malignant mesothelioma cells (HM) as a model for a ROS-driven tumor cell line [21] we sought to investigate the phenotype associated with lowering expression of the mitochondrial oxidoreductase peroxiredoxin 3 (PRX3), the antioxidant enzyme responsible for metabolizing the majority of mitochondrial peroxide [22]. HM cell lines stably expressing short hairpin RNA against PRX3 were generated (HMshPRX3) and successful knockdown was confirmed by immunoblotting (Fig. 1A) and immunofluorescence microscopy (Fig. 1B). The relative mean fluorescence intensity (MFI) for anti-PRX3 signal was quantified from immunofluorescence images and found to be approximately 6-fold lower than HM cells (Fig. 1C). PRX3 is estimated to metabolize approximately 90% of mitochondrial H_2_O_2_ [22] and therefore it would be expected that reduced expression of PRX3 would alter the redox status of the mitochondria. HM and HMshPRX3 cells were loaded with MitoSOX Red, a fluorescent indicator of general oxidant levels in the mitochondrial matrix, and analyzed by flow cytometry. HMshPRX3 cells showed a 30% increase in MitoSOX RED fluorescence, thereby indicating an increase in the levels of mitochondrial oxidants in these cells (Fig. 1D).

### Knockdown of PRX3 alters the metabolic profile of HM cells

**Fig. 2:**
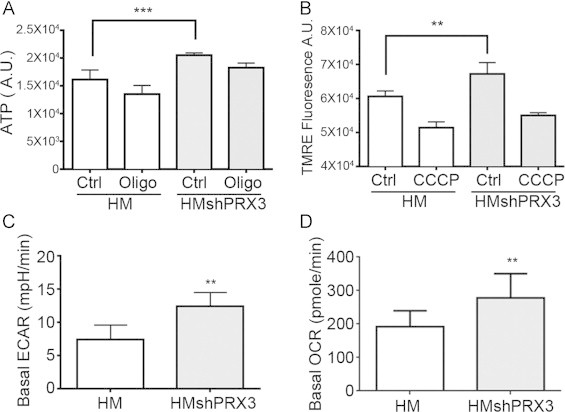
HMshPRX3 cells have an altered metabolic phenotype. (A) ATP levels (A.U.=arbitrary units) measured in HM and HMshPRX3 cells. 5 µM oligomycin (Oligo) reduces ATP levels by blocking complex V activity (^⁎⁎⁎⁎^*p*<0.0001, *n*=5). (B) Mitochondrial membrane potential measured by TMRE fluorescence in HM and HMshPRX3 cell lines. 1 µM CCCP uncouples mitochondrial membrane potential (^⁎⁎^*p*<0.01, *n*=5). (C) Basal extracellular acidification rates (ECAR) of HM and HMshPRX3 cells measured with a seahorse Bioanalyzer (^⁎⁎^*p*<0.01, *n*=5). (D) Basal oxygen consumption rates (OCR) of HM and HMshPRX3 cells (^⁎⁎^*p*<0.001, *n*=5).

Among the many processes of mitochondria, including calcium buffering [23], propagation of apoptotic signals [24] and production of ROS [4], their primary role is to provide energy in the form of adenosine triphosphate (ATP). The transport of electrons through the electron transport chain (ETC) with oxygen as the final electron acceptor supports the pumping of hydrogen atoms across the inner mitochondrial membrane, thereby producing the electrochemical gradient that drives ATP production at complex V (ATP synthase). We measured ATP levels in HMshPRX3 cells and found them to be significantly elevated above those of HM cells, and both cell lines were responsive to the ATP synthase inhibitor oligomycin (Fig. 2A). Mitochondrial membrane potential supports the flow of electrons through the ETC, and ATP levels fluctuate based on changes to mitochondrial membrane potential. Supporting the evidence that HMshPRX3 cells have increased relative ATP levels as compared to HM cells, they also have an increased mitochondrial membrane potential compared to HM cells as measured with the cell permeant positively charged ethyl ester TMRE (Fig. 2B).

Using Seahorse extracellular flux analysis we compared metabolic features of HM and HMshPRX3 cells. Basal oxygen consumption rates (OCR) and extracellular acidification rates (ECAR) were increased in HMshPRX3 cells compared to HM cells (Fig. 2C and D). The observed increase in ATP levels, membrane potential and oxygen consumption suggests increased mitochondrial metabolism in HMshPRX3 cells. We could not directly measure this change as HMshPRX3 cells failed to respond to ETC inhibitors to reveal reproducible mitochondrial metabolism profiles using the Seahorse Bioanalyzer (data not shown, see discussion). To control for differences in mitochondrial mass, which would confound the observed metabolic changes, mitochondrial mass was quantified from fluorescence images and found to be equal for both the shPRX3 and HM control cell lines (Supplemental Fig. 1).

### Knockdown of PRX3 induces mitochondrial fusion and cell cycle arrest in G2/M

**Fig. 3:**
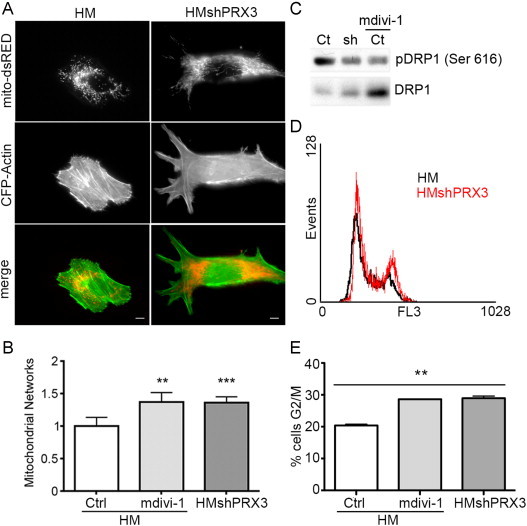
HMshPRX3 cells exhibit hyperfused mitochondrial networks and altered cell cycle phenotype. (A) Fluorescent images of HM and HMshPRX3 cells transfected with expression vectors for mito-dsRED and CFP-Actin. Scale bar=10 µm. (B) Quantification of mitochondrial networks (see Materials and methods section) in control (Ctrl), mdivi-1 treated HM cells and HMshPRX3 cells (^⁎⁎^*p*<0.01, ^⁎⁎⁎^*p*<0.001, *n*=10 cells). (C) Immunoblot for phospho-DRP1 (Ser 616) and total DRP1 in HM (Ct), HMshPRX3 (sh) and HM cells treated with 10 µM mdivi-1 for 24 h (D) representative cell cycle flow cytometry histograms of HM and HMshPRX3 cells stained with propidium iodide. (E) Percentage of cells in the G2/M cell cycle phase in the indicated cell lines (^⁎⁎^*p*<0.01, *n*=3).

Mitochondrial structure is affected by and directly influences numerous functional processes, including ATP production [16,19], ROS production [25,26] and calcium buffering [27]. Given the significant metabolic differences between HMshPRX3 and HM cells we investigated if there were differences in mitochondrial networks. HM and HMshPRX3 cells were transfected with fluorescent expression vectors to label mitochondria (mito-dsRED) and the actin cytoskeleton (CFP-Actin). HM cells showed a fragmented perinuclear mitochondrial network (Fig. 3A), reminiscent of other tumor cell types in which mitochondrial fission supports tumor cell migration and survival [28,29]. Conversely HMshPRX3 cells showed a more elongated and filamentous mitochondrial network that extended into the cell periphery (Fig. 3A). HMshPRX3 cells were observed to be slightly larger than HM cells but no statistically significant increases in cell mass were observed (data not shown). Quantification of mitochondrial networks showed that HMshPRX3 cells exhibited a significant increase in mitochondrial length and branching, which could be recapitulated in HM cells by incubation with the DRP1 fission inhibitor mdivi-1 (Fig. 3B).

DRP1 has two previously described phosphorylation sites that differentially regulate its activity [30,31]. Phosphorylation at serine 616 of DRP1 leads to increased activity and mitochondrial fission. HMshPRX3 cells showed decreased phosphorylation of DRP1 at serine 616 similar to that of HM cells treated with the DRP1 inhibitor mdivi-1 (Fig. 3C), and this change correlated with increased mitochondrial networking observed by fluorescence imaging (Fig. 3A and B).

Mitochondria undergo fission at late metaphase prior to mitosis in a DRP1-dependent manner, which ensures equal distribution of mitochondria to daughter cells [32]. Given the mitochondrial structural aberrations and reduced DRP1 phosphorylation at serine 616 in HMshPRX3 cells, we investigated cell cycle progression in HMshPRX3 cells compared to HM controls. A representative cell cycle histogram as analyzed by flow cytometry after propidium iodide staining indicated HMshPRX3 cells have an increased proportion of cells in the G2/M cell cycle phase compared to HM cells (Fig. 3D). Comparison of the percentage of cells in G2/M to G1 and S phases of the cell cycle showed that HMshPRX3 cells had significantly more cells in the G2/M phase compared to HM cells (Fig. 3E). An increase in cells in the G2/M cell cycle phase was also seen in HM cells treated with the DRP1 inhibitor mdivi-1 (Fig. 3E). These data indicate that mitochondrial fusion in HMshPRX3 cells contributes to an accumulation of cells at the G2/M phase of the cell cycle.

### Catalase expression rescues mitochondrial structural aberrations and cell cycle abnormalities in HMshPRX3 cells

**Fig. 4:**
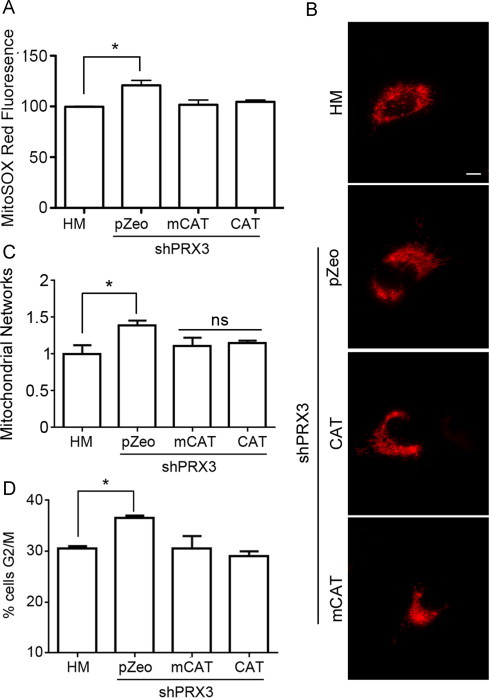
Catalase expression restores HMshPRX3 mitochondrial structural aberrations and cell cycle abnormalities. (A) Mitochondrial oxidant levels in cells transfected with indicated expression vectors. MitoSOX Red oxidation was measured by flow cytometry 24 h after cell transfection (^⁎^*p*<0.05, *n*=2). (B) Mitochondria of HM cells and shPRX3 cells transiently transfected with control vector (pZeo), catalase (CAT), or mito-catalase (mCAT) and mito-dsRED were imaged by fluorescent microscopy 24 h after transfection (scale bar = 10 µm). (C) Quantification of mitochondrial networks of cells transfected with the indicated expression vectors as in A (^⁎^*p*<0.05, n.s.=not significant, *n*=10 cells). (D) Percentage of cells in the G2/M cell cycle phase as measured by flow cytometry in cells transfected with indicated expression vectors as in A (^⁎^*p*<0.05, *n*=3).

Increased oxidant levels disrupt normal cell cycle progression and have been shown to induce mitochondrial fusion as a means of escaping increased oxidative stress [33]. To determine whether increased oxidant levels contribute to the altered mitochondrial structure and abnormal cell cycle profiles observed in HMshPRX3 cells, we expressed the H_2_O_2_ scavenger catalase (CAT) or a mitochondrial targeted catalase (mCAT) in HMshPRX3 cells and examined mitochondrial oxidant levels, mitochondrial structure and cell cycle progression. Expression of CAT or mCAT in HMshPRX3 cells significantly lowered the level of mitochondrial oxidants to the levels comparable to HM cells as measured by flow cytometry after staining with MitoSOX Red (Fig. 4A). Analysis of fluorescent images of cells expressing mito-dsRED to label mitochondria showed that HMshPRX3 cells expressing CAT or mCAT showed a more fragmented mitochondrial network than HMshPRX3 cells expressing control vector (Fig. 2B). Quantification of mitochondrial length and branching confirmed this observation as HMshPRX3/CAT and HMshPRX3/mCAT cells had mitochondrial networks similar to HM cells, while HMshPRX3 cells continued to harbor fused mitochondrial networks (Fig. 4C).

We next investigated if the lower mitochondrial redox status and fragmented mitochondrial network were sufficient to rescue the G2/M cell cycle block observed in HMshPRX3 cells. HMshPRX3/CAT and HMshPRX3/mCAT cell cycle profiles were determined by flow cytometry after propidium iodide staining, and showed a reversal of the G2/M cell cycle block observed in HMshPRX3 cells (Fig. 4D).

## Discussion

Our data support the hypothesis that PRX3 establishes a redox set point required for proper mitochondrial structure and cell cycle progression. Increases in mitochondrial oxidants and changes in antioxidant expression have been implicated in a wide range of pathological states where altered redox balance contributes to the onset and progression of the disease [34]. Specifically, mitochondrial oxidants are increased in cancer, including KRAS driven lung tumors, and support proliferation and metastasis of tumor cells [35]. Changes in the redox landscape of tumor cells activates stress response programs that would typically lead to senescence unless excessive oxidants are counterbalanced through metabolic reorganization, as for example by up-regulation of antioxidant enzymes [36]. The redox responsive oncogenic transcription factor FOXM1 is expressed in every solid tumor studied to date and is required for escape from cytotoxic oxidant levels by increasing the expression mitochondrial antioxidant enzymes SOD2 (MnSOD) and PRX3 [3]. This adaptation is required for oncogenic transformation and has been shown to be a unique metabolic target as it is largely independent of any specific oncogene or tumor suppressor alterations [36].

Increased expression of peroxiredoxins, including PRX3, in tumor cells counterbalances excessive cellular oxidants and promotes a redox-dependent pro-proliferative state [3]. This pro-proliferative state requires proper cell cycle progression, which is intimately linked to cellular redox status [37,38], with the earliest reports from the 1930s that indicated variability in protein thiol status during different cell cycle phases [39]. Extensive studies since then have shown fluctuations in antioxidant expression levels, GSH:GSSG ratios and oxidant levels during different cell cycle phases [40]. The mitochondria, being a primary intracellular source of oxidants, have also been implicated as an important source of oxidants that govern cell cycle progression [41]. The activity of the mitochondrial superoxide scavenger MnSOD has been shown to modulate cell cycle entry, exit and progression [42], while we have reported significant increases in mitochondrial oxidants during progression from G1 to M phase [43].

The data reported here support a role for PRX3 in the regulation of cell cycle dynamics (Figs. 3 and 4), while others have shown the role of other PRX family members in this process. H_2_O_2_-dependent cell cycle arrest leads to the hyperoxidation of PRX2, inducing the formation of higher molecular weight oligomers, which must be reduced prior to cell cycle reentry [44]. Knockdown of PRX2 in glioblastoma cells leads to arrest in G2/M with a concomitant increase in intracellular and extracellular oxidants [45]. Inactivation of PRX1 through phosphorylation by CDK1 (Cdc2) stimulates transient, localized H_2_O_2_ levels hypothesized to be required for inactivation of the protein tyrosine phosphatase Cdc25C and the complete activation of CDK1 at mitosis [46]. Using a novel bioinformatics approach, Conour et al. [47] proposed that the majority of redox-responsive cell cycle proteins are involved in regulating transition through G2/M.

We show here that knockdown of PRX3 using targeted shRNAs in malignant mesothelioma cells (HMshPRX3) led to an increased mitochondrial oxidation state that was reversible through the over-expression of catalase or mito-catalase (Fig. 4). Mitochondria dynamically alter their structure and distribution in response to intracellular signals including Ca^2+^ levels, energy needs and stress. Mitochondrial structural rearrangements are coupled to functional aspects of mitochondrial signaling and metabolism [48]. Our data indicate that mitochondrial redox status influences metabolic features of mitochondria as HMshPRX3 cells exhibit increased mitochondrial membrane potential and ATP levels (Fig. 2). This is in agreement with other studies that show reversible cysteine modifications to mitochondrial ETC proteins regulate their activity and overall metabolic state [49]. The requirement of mitochondrial metabolic demands to be responsive to oxidative stress is supported by the general finding that ATP levels and mitochondrial ROS exhibit an inverse relationship.

Using Seahorse extracellular flux analysis we show that HMshPRX3 cells have increased oxygen consumption rates (OCR) and extracellular acidification rates (ECAR) as compared to vector WT controls (Fig. 2). HM tumor cells have a reduced mitochondrial reserve capacity and a higher demand for mitochondrially-derived ATP as compared to immortalized non-tumorigenic mesothelial cells, while having similar ECAR profiles (manuscript in revision). We were unable to confirm the mitochondrial reserve capacity of HMshPRX3 cells or their respective demand for mitochondrial ATP as compared to WT controls because after addition of oligomycin OCR rates dissipated and maximal respiration could not be stimulated with the addition of CCCP (data not shown). These results may indicate that the structural and metabolic rearrangements to the mitochondria seen in HMshPRX3 cells reorganizes their metabolic demand to be more reliant on mitochondrial ATP. HMshPRX3 cells also had an increased ECAR compared to WT controls, suggesting knockdown of PRX3 increases glycolysis (Fig. 2). Changes in cellular oxidants such as have been shown to regulate glycolytic pathways; specifically increasing ROS stimulates glycolysis, while in contrast overexpression of superoxide dismutase (SOD) reduced glycolysis independent of hypoxia in hepatocytes [50]. MM cells are more sensitive to changes in oxidant levels as compared to immortalized non-tumorigenic and primary mesothelial cells, and have distinctive metabolic demands (manuscript in revision). Therefore further investigation will be needed to understand the role of mitochondrial H_2_O_2_ in the regulation of glycolytic pathways, particularly in the survival and proliferation of rapidly dividing cancer cells.

Mitochondrial fission is required prior to cytokinesis to ensure proper distribution to daughter cells, whereas mitochondrial hyperfusion supports increased ATP levels and cyclin E expression required for G1-S progression. Increased mitochondrial stress has been shown to induce fusion of organelles and the pro-fusion regulator mitofusin-2 (MFN2) is redox-regulated through reversible cysteine oxidation that promotes mitochondrial fusion [51]. Analysis of mitochondrial structure in HMshPRX3 cells showed an increase in mitochondrial networking as compared to wild type controls (Fig. 3). Although we did not investigate the activity or oxidation state of MFN2 in these cells we did identify post-translational changes in the primary fission molecule DRP1. Phosphorylation of DRP1 at serine 616 promotes mitochondrial fission, and we observed a significant reduction in phosphorylation levels of serine 616 of DRP1 in HMshPRX3 cells (Fig. 3). This loss in phosphorylation was also seen in wild type cells treated with the DRP1 inhibitor mdivi-1 that also promoted mitochondrial networking (Fig. 3).

Mitchel et al. have recently reported that endoplasmic reticulum stress in Akita beta cells with mutations to the B insulin chain (Akita +/Ins2) have increased mitochondrial oxidative stress and concomitant mitochondrial network fragmentation [52]. Although these and other studies [25,26] indicate mitochondrial oxidative stress is associated with mitochondrial fragmentation it is difficult to compare results across cells lines. Basal redox status, metabolic requirements and different genetic signatures no doubt influence mitochondrial stress responses. Other groups have identified hyperfused mitochondrial networks during autophagy [15], a process that is stimulated by mitochondrial-derived oxidants [53]. These latter results indicate, as for other cellular stress responses, that the length and duration of oxidant signaling influence mitochondrial responses. A better understanding of the molecular pathways that integrate specific redox modifications with diverse structural and functional processes (e.g. mitochondrial fission–fusion cycles and metabolism) will be required to clarify these apparently contradictory findings.

Based on our results we propose a model in which increased mitochondrial oxidative stress caused by knockdown of PRX3 induces mitochondrial hyperfusion through the inactivation of DRP1. The altered redox status and increased mitochondrial networking impairs proper cell cycle progression, perhaps by prolonging transition through G2/M. Ectopic expression of catalase or mito-catalase rescued the phenotypic effects caused by diminished expression of PRX3, implying that increased levels of H_2_O_2_ were responsible for the phenotypic effects of knocking down PRX3. We suggest that PRX3 establishes a mitochondrial redox set point required for proper mitochondrial structure, function and cell cycle progression. Future work will be needed to fully understand the link between mitochondrial redox status, mitochondrial structure, energy metabolism and cell cycle progression, and how these processes are corrupted during neoplastic transformation to promote tumorigenesis.

## Materials and methods

### Cell lines and cell culture

Human malignant mesothelioma cells (HM) were used in this study [54]. Cells were maintained in DMEM-F12 with hydrocortisone, insulin, transferrin, and selenite with 10% fetal bovine serum (FBS; GIBCO, Grand Island, NY).

### Generation of stable Ctrl and shPRX3 cell lines

PRX3 and PLKO.1 lentiviral shRNAs were purchased from Sigma and packaged following the manufacturers protocol (Sigma). Stable shPRX3 and PLKO.1 (HM) cell lines were established by seeding 35 mm tissue culture dishes with 1.25×105 HM cells and allowed to adhere overnight. The next day 150 µL of medium containing shPRX3 lentiviral particles or shCtrl particles were added to cells for 18 h. The cells were washed and incubated with complete media to remove viral particles. Following 2 days, culture media was replaced with complete media containing µg/mL--2 µg/mL puromycin and subsequently changed every 2 days until control non-transduced cells were completely non-viable.

### Cell cycle analysis

To determine cell cycle profiles cells were washed 1× with PBS, trypsinized, and resuspended in 1× PBS. Cells were fixed by adding cold 100% EtOH drop wise while vortexing to a final concentration of 80% EtOH. After incubation for 30 min at 4 °C, EtOH was removed by centrifugation and cells were resuspended in a working stock of propidium iodide (PI) containing RNase and 1% fetal calf serum. Cell cycle profiles were determined by flow cytometry.

### Transient transfections

Cells were plated into 35 mm dishes and transfected the following day with expression vectors for pZeo (control), catalase, or mito-catalase using Lipofectamine 2000 (Invitrogen) following the manufacture's instructions. For mitochondrial morphological assessment and transfection efficiency mito-dsRED was included in the transfection. Cells were used 24 h after transfection for indicated experiments.

### Reagents and inhibitors

Mdivi-1 was purchased from Enzo Life Sciences (Farmingdale, NY) and tetramethylrhodamine ethyl ester (TMRE) was purchased from Invitrogen. Oligomycin A and carbonyl cyanide m-chlorophenyl hydrazine (CCCP) were purchased from Sigma (St. Louis, MO). All compounds were re-suspended in molecular grade sterile dimethyl sulfoxide (DMSO).

### Immunoblotting

Cell lysates were prepared by washing cells 1× with PBS and scraping in NP-40 lysis buffer. After centrifugation at 14,000 RPM protein concentrations were determined using Bradford assays (Bio-Rad, Hercules, CA). Lysates (10–15 µg protein/well) were resolved by reducing and denaturing polyacrylamide gel electrophoresis (SDS-PAGE) and prepared for immunoblotting as previously described [23]. Blots were blocked with 5% non-fat milk for 1 h. and incubated at 4 °C overnight with rabbit anti-PRX3 polyclonal antibody (Ab Frontier, Seoul, Korea) at a 1:2000 dilution, anti-pDRP1 (Serine 616, Cell Signaling) at a 1:500 dilution, or anti-DRP1 (Cell Signaling) at a 1:1000 dilution in blocking buffer. Membranes were washed the following day 5× for 5 min with 1× Tris-buffered saline containing 0.2% Tween-20 (TBST). After washing, protein bands were visualized with the Western Lightning chemiluminescent detection system (PerkinElmer, Waltham, MA) using secondary antibodies coupled to horse radish peroxidase. Blots were stripped and re-probed with a mouse anti-actin antibody (Millipore, Billerica, MA) to verify equal protein loading.

### MitoSOX Red flow cytometry

Cells were loaded with 5 µM MitoSOX Red (Invitrogen) in tissue culture medium for 30 min. Cells were washed with Hanks buffered salt solution with calcium and magnesium (HBSS), collected by brief trypsinization, centrifuged, and washed twice in HBSS. Cell pellets were re-suspended in 1% bovine serum albumin (BSA) in HBSS, and approximately 10,000 events were collected by flow cytometry. To measure oxidized MitoSOX Red, cells were excited at 488 nm and emission was collected in the FL2 channel.

### ATP assay

ATP levels were determined using the ATPlite assay kit from PerkinElmer (Waltham, MA). 2500 cells per well were plated into 96-well plates and treated the following day with indicated compounds for 1 h. After treatment, cells were washed 1× with PBS and 50 µL of PBS was added back to each well. 25 µL of cell lysis solution was added to each well and the plate was shaken on an orbital shaker for 5 min 25 µL of ATP substrate was added to each well and mixed. 75 µL from each well was transferred to a white 96-well plate. The plate was subsequently dark adapted for 10 min and luminescence was read in a Synergy HT plate reader (BioTek, Winooski, VT).

### Measurement of mitochondrial morphology

HM and HMshPRX3 cells were transfected with plasmids encoding Mito-dsRED and Cyan-LifeAct, as previously described [43], to visualize mitochondria and actin cytoskeleton by fluorescence microscopy, respectively. Images were collected on a Nikon Ti-E inverted microscope using the TRITC and CFP filter sets at appropriate exposure times using a 60× objective. Images of mitochondria were subjected to top-hat filtering as previously described [55] using ImageJ software (NIH). The area and perimeter of mitochondrial particles were determined and used to calculate a mitochondrial form factor (FF), a measure of mitochondrial length and branching, using the following formula: FF=perimeter^2^/4*π*×area. Data are expressed as percent mitochondrial networks compared to control cells.

### Measurement of mitochondrial membrane potential

Cells were plates in 96-well plates at a density of 2500 cells per well and treated the following day with indicated compounds for 1 h. 10 µM carbonyl cyanide m-chlorophenyl hydrazone (CCCP) was added to specific wells and incubated for 10 min. The culture media was removed and cells were incubated with 250 nM tetramethylrhodamine, ethyl ester (TMRE). The plate was incubated for 10 min. The cells were washed 1× with PBS and each well was re-suspended in 100 µL PBS+0.2% BSA and fluorescence (ex 530 nm/em 590 nm) was read in a plate reader, as described above.

### Oxygen consumption rates (OCR) and extracellular acidification rates (ECAR)

Cells were plated in 24-well XF24 cell culture microplates at a density of 40,000 cells per well. The following day cells were washed 1× with XF cell culture media and 560 µL was added back to each well. Cells were allowed to equilibrate in a CO_2_ independent incubator for 1 h. Basal OCR and ECAR were determined using an XF24 Seahorse Bioanalyzer (Seahorse Bioscience, North Billerica, MA). Oxygen and proton concentrations were measured 3× over a 17 min period and graphed as the mean of 5 replicates.

### Statistical analysis

Data are presented as ±SEM. Statistical significance was determined using 1-way ANOVA with a Tukey's post-hoc test or the students *t* test comparing control to experimental conditions for *p*<0.05.

## Appendix A Supplementary materials

Supplementary Fig. 1 Mitochondrial mass in HM and HMshPRX3 cells. Quantification of mitochondria in HM and HMshPRX3 cells expressing mito-dsRED and CFP-Actin. Mean gray values for the mito-dsRED signals were quantified using ImageJ and normalized to cell area to show relative mitochondrial amounts in each respective cell line (*n*=10 cells).
