# Supplementary figures and images for: Peroxiredoxin 3 levels regulate a mitochondrial redox setpoint in malignant mesothelioma cells
Source: Redox Biol. 2014 Nov 18;3:79–87. doi: 10.1016/j.redox.2014.11.003 (PMC4297934; doi:10.1016/j.redox.2014.11.003)

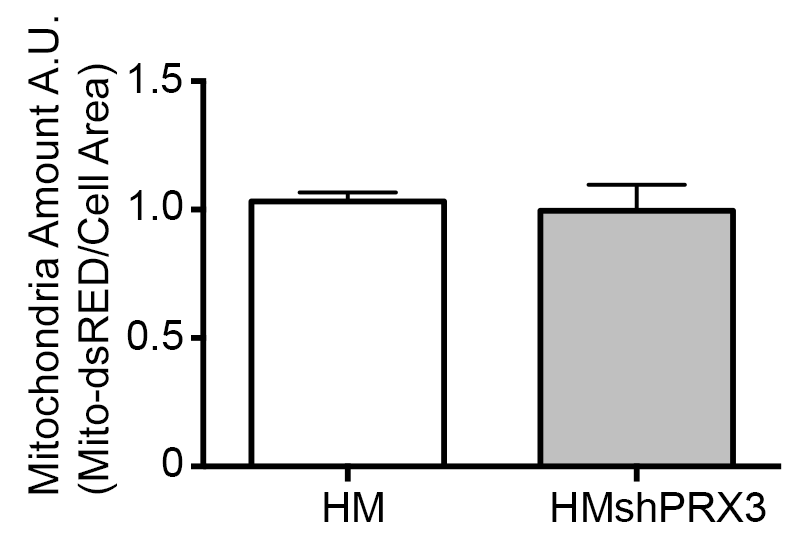

Supplement: Supplementary Fig. 1 — Mitochondrial mass in HM and HMshPRX3 cells. Quantification of mitochondria in HM and HMshPRX3 cells expressing mito-dsRED and CFP-Actin. Mean gray values for the mito-dsRED signals were quantified using ImageJ and normalized to cell area to show relative mitochondrial amounts in each respective cell line (n=10 cells). [file mmc1.zip › Supplemental Figure 1.tif]
